# Clinical Genetics in Japan: Efforts of Human Genetics Societies and Related Organizations

**DOI:** 10.31662/jmaj.2019-0019

**Published:** 2019-09-10

**Authors:** Yoshimitsu Fukushima, Fumio Takada

**Affiliations:** 1 Department of Medical Genetics, Shinshu University School of Medicine, Matsumoto, Japan; 2 Department of Medical Genetics and Genomics, Kitasato University Graduate School of Medical Sciences, Sagamihara, Japan

**Keywords:** Genomic Medicine, Clinical Genetics, Grassroot movements of academia

## Abstract

The Japanese government finally started measures to promote the realization of genomic medicine that can promote the accumulation of individual genomic information for improving medical care in 2015. However, readiness in terms of social infrastructure (including legal, administrative, ethical, and educational aspects in Japan) remains inadequate. Associations related to medical genetics have been making consistent efforts to realize genomic medicine by establishing guidelines, nurturing genetic professionals, providing support for constructing cross-disciplinary medical systems, enriching genetic education, etc., and it is important that the Japanese government supports these initiatives.

## Introduction

On January 20, 2015, Barack Obama, the former President of the United States of America, promoted the precision medicine initiative (PMI) as a measure for science and technology in the State of the Union Address ^(1)^. PMI is a research effort to further advance initiatives for connecting individual genomic information conventionally referred to as personalized medicine. Consequently, the promotion of the realization of genomic medicine is now regarded as one of the national policies in Japan ^(2)^.

However, genomic information includes germline-genetic information, which is life-long invariance, and it is possible to predict the onset of illness with a considerable degree of probability. Moreover, there is a possibility that germline-genetic information is related not only to the health of relevant individuals, but also to that of consanguineous relatives. Therefore, it is important to develop a social system to enrich the genetic education level to eliminate and prevent misunderstandings, prejudice, and discrimination with respect to the application of genome information in clinical practice.

Peter S. Harper, a prominent British medical geneticist, has described medical genetics in Japan in his book entitled “A short history of medical genetics” as follows ^(3)^:

“Japan provides an unusual situation, for medical and human genetics have here been particularly weak, despite highly developed scientific, technological, and medical traditions. Mendelian genetics was taken up very early in Japan for the purpose of plant breeding, while after World War II radiation genetics and human cytogenetics grew strongly in the wake of the atomic bomb disasters and consequent research. Cultural isolation and extreme sensitivity over family matters, including genetic disorders, may have been delaying factors for medical genetics, as may the fact that much research on genetic disorders has been channeled through other medical specialties. A thorough study of all these aspects is needed.”

We have no choice but to accept his message that genetic education and medical genetics in Japan is extremely lagging in comparison with those in advanced countries, such as Europe and the United States. However, we, Japanese physicians, researchers, and members of associations related to medical genetics in Japan, have been exploring and practicing the establishment of medical genetics and genomic medicine systems that can be applied to the Japanese culture and society. In particular, since the initiative to promote the realization of genomic medicine became a national policy of Japan in 2015, the establishment of medical genetics and genomic medicine systems “from research to clinical practice” has gained momentum. Here we have mainly highlighted the initiatives of Japanese associations related to medical genetics that have also been cited in the documents of the Council for the Promotion of the Realization of Genomic Medicine ^(2)^.

## I. Associations Related to Medical Genetics in Japan

### 1) The Japan Society of Human Genetics ^(4)^

In 1956, The Japan Society of Human Genetics (JSHG) was established independent of The Genetics Society of Japan by researchers and physicians interested in human genetic phenomena. The JSHG develops technologies to elucidate human genetic phenomena and includes people from diverse research backgrounds, including cytogenetics, biochemistry, molecular genetics, embryology, oncology, pediatrics, obstetrics and gynecology, neurology, otology, etc. as its members; the society includes 5,112 members as of January 2019.

The JSHG was established with the aim of contributing to the advancement of science via human genetic studies, and was included as a member of The Japanese Association of Medical Sciences in 1964. The various activities of the JSHG, including disseminating knowledge on human genetics via educational and awareness programs, have been conducted to promote medical care in the field of genetics. In particular, considering the diversity among individuals is becoming more evident, the JSHG not only plays the leading role through guiding principles and guidelines for research and medical care, but is also actively engaged in educational and social awareness activities to build a society based on mutual respect for human diversity, thereby ensuring no discrimination regarding individual differences, which are described in the website (Philosophy and future plans of the JSHG) ^(5)^.

In 2016, the 13th International Congress of Human Genetics (ICHG), hosted by the JSHG, was held at Kyoto. There were 3,306 participants from 70 countries, of whom 1,280 people were from abroad and 2,026 people were from Japan. The ICHG has been held once every five years since 1956, but this is the first time it has been held in Asia, and it is very pleasing that Japan could contribute to the development of human genetics and genomic medicine ^(6)^.

The contribution of the JSHG to human genetics is extremely significant, as noted from the diseases identified by members of the JSHG (Table 1) ^(6)^. The JSHG also publishes two academic journals: Journal of Human Genetics ^(7)^ and Human Genome Variation ^(8)^. Furthermore, the JSHG collaborates with related associations and groups and is actively making consistent efforts to realize genomic medicine by establishing guidelines, nurturing genetics professionals (including clinical geneticists, clinical cytogeneticists, and certified genetic counselors), and enriching genetics education.

**Table 1.** Identified Pathogenic Genes and New Disease Entities by Japanese Researchers. (Reported by Yoichi Matsubara in ICHG 2016 ^(6)^. Reproduced with Permission.)

Amyotrophic lateral sclerosis (OPTN)
Argininemia (ARG1)
AR Spinocerebellar ataxia (SYT14)
Axial spondylometaphyseal dysplasia (C21orf2)
Beta ketothiolase deficiency (ACAT1)
BH4-responsive phenylketonuria (PAH)
Camurati-Engelmann disease (TGFB1)
Charcot-Marie-Tooth disease type 1B (MPZ)
Chediak-Higashi syndrome (LYST)
Citrin deficiency (SLC25A13)
Coffin-Siris syndrome (SWI/SNF genes, SOX11)
Congenital insensitivity to pain with anhidrosis (NTRK1)
DRPLA (ATN1)
Ehlers-Danlos syndrome, Kosho type (CHST14)
Familial polyposis/colon cancer (APC)
Fukuyama congenital muscular dystrophy (FKTN)
Glycosylation disorder (COG2, PIGG)
HCAHC (POLR3A, POLR3B)
Hypospadias (MAMLD1 (CXorf6) )
Hypothalamic hamartomas (OFD1, GLI3)
Kabuki syndrome (KDM6A)
Kagami-Ogata syndrome (UPD(14)pat)
Leigh syndrome (GYG2)
Mabry syndrome (PIGL)
Machado-Joseph disease (ATNX3)
Marfan syndrome type 2 (TGFBR2)
MELAS syndrome (MT-TL1)
Microphthalmia syndrome (SMOC1)
Morquio syndrome (GALNS)
Moyamoya disease (RNF213)
Multiple carboxylase deficiency (HLCS)
Nemaline myopathy (KLHL40)
Nijmegen breakage syndrome (NBS1)
Non-ketotic hyperglycinemia (GLDC, AMT, GCSH)
Ohtahara syndrome (STXBP1, KCNQ2, CASK, GNAO1）
Peroxisomal disorders
Porencephaly (COL4A2)
Primary systemic carnitine deficiency (SLC22A5)
Prolidase deficiency (PEPD)
Radioulnar synostosis with amegakaryocytic thrombocytopenia (MECOM)
RASopathies
Costello syndrome (HRAS)
CFC syndrome (KRAS, BRAF)
Noonan syndrome (RIT1)
Schizencephaly (COL4A1)
Segawa syndrome (TH)
SEMD-JL1 (B3GALT6)
SENDA (WDR45)
Sotos syndrome (NSD1)
Spinal extradural arachnoid cyst (HOXD4)
Steroid-resistant nephrotic syndrome (NUP107)
Takenouchi-Kosaki syndrome (CDC42)
Tarui disease (glycogen storage disease, type VII)(PFKM)
Tyrosinemia type III (HPD)
Very long-chain acyl-CoA dehydrogenase deficiency (ACADVL)
West syndrome (SPTAN1)
Xeroderma pigmentosum, group A (XPA)

### 2) The Japanese Society for Genetic Counseling ^(9)^

The Japanese Society for Genetic Counseling (JSGC) aims to promote the advancement, development, and dissemination of clinical genetics in Japan and to contribute to the improvement of medical care and welfare through the practice of clinical genetics research and fair genetic counseling in response to its extensive demand among the public. The former society (The Japan Society of Medical Genetics) was renamed in 2001. As of January 2019, the JSGC has 1,617 members, including physicians, nurses, certified genetic counselors and other medical professionals. The society manages an accreditation system for clinical geneticists and certified genetic counselors collectively along with the JSHG.

### 3) The Japanese Society for Gene Diagnosis and Therapy ^(10)^

The Japanese Society for Gene Diagnosis and Therapy (JSGDT) aims to gather members who are active in other associations of medical genetics and in associations related to laboratory medicine. The JSGDT also aims to promote and improve research on the clinical applications of gene-related technologies. It is important to connect research in medical and clinical genetics in association with laboratory medicine and to solve technical issues related to information processing and other aspects of regulatory science that constitute the basis for promoting genetic practice in the medical field. An attribute of the JSGDT is that not only stakeholders in various positions in academia, but also those involved in clinical testing, in vitro diagnostics manufacture, medical equipment manufacture, pharmaceutical companies, and regulatory affairs gather to provide practical information and hold discussions.

## II. Human Resource Development and the Accreditation System

### 1) The Japanese Board of Medical Genetics and Genomics, Clinical Genetics (JSHG and JSGC, 1991-) ^(11)^

The Japanese Board of Medical Genetics and Genomics, Clinical Genetics responds to consultations from all clinical departments of the hospital and conducts appropriate genetic medical care while also acting as an association that resolves genetics-related issues that are expected to occur in each medical institution. The Board seeks the following abilities:

a. To possess extensive specialized knowledge regarding medical genetics,

b. to conduct specialized tests, establish diagnosis, and provide treatment in specific domains in the field of medical genetics,

c. to conduct genetic counseling,

d. to possess adequate knowledge regarding and experience in genetic testing, and

e. to possess adequate achievements in medical genetics research and be able to implement education in medical genetics.

After gaining specialization in a fundamental domain and undergoing specialized training in clinical genetics for more than three years, medical doctors (MDs) can attain eligibility requirements for examinations. As of January 2019, 1,388 MDs have been certified.

### 2) The Japanese Board of Medical Genetics and Genomics, Clinical Cytogenetics (JSHG, 1994-) ^(12)^

The JSHG promotes the appropriate implementation of chromosome testing in medical care in Japan by physicians, researchers, and technologists involved in chromosome analysis in clinical testing. The Japanese Board of Medical Genetics and Genomics, Clinical Cytogenetics was established in 1994 with the aim of improving the accuracy of techniques for chromosome testing and of further developing clinical cytogenetics.

The Board is specialized in accurately determining the chromosome testing results based on the profound knowledge of advanced technologies in medical genetics. As of April 2018, 178 individuals have been certified in this aspect.

### 3) Certified Genetic Counselor system (JSHG and JSGC, 2005-) ^(13)^

Although training non-physician genetic counseling personnel has been challenging for a long time, the certified genetic counselor system was established in 2005. Currently, it is possible to meet the eligibility requirements for certified genetic counselor examinations by receiving full-time education and training in 16 graduate school master’s programs that have been accredited by the system committee. Certified genetic counselors are expected to possess the following abilities:

a. Up-to-date knowledge on medical genetics,

b. specialized counseling techniques, 

c. potential to deal with ethical–legal–social issues, and

d. potential to build and maintain collaborative relations (team) with physicians and other medical professionals.

As of January 2019, 243 people have received this certification and the system has become the center of medical genetics in Japan that works in collaboration with clinical genetics specialists in various domains, including departments of medical genetics in university hospitals, cancer treatment core hospitals, prenatal care facilities, and medical educational institutions.

### 4) Genetic Expert system (JSGDT, 2015-) ^(14)^

The Genetic Expert System is a new training system for human resources that was started in 2015 by the JSGDT. Genetic experts are specialists in gene diagnosis and therapy and are involved in selecting appropriate information and accurately interpreting the results of human gene-related tests and genetic information, including germline genetic tests and somatic cell genetic tests. They participate in quality control and are also engaged in quickly and clearly reporting and explaining the significance of the results to medical personnel. They can also play the leading role in developing inspection methods based on databases. The eligibility criteria for genetic experts include work experience of > 3 years in facilities related to genetic testing and participation in a clinical genetics information retrieval course. As of 2018, 27 people are certified genetic experts.

## III. History of the Establishment of the Clinical Genetics Medical Care System

In Japan, the development of the clinical genetics medical care system was extremely delayed compared to the progress of basic medical genetics research. The first Japanese medical genetics division was established in the Department of Genetics, Kanagawa Children’s Medical Center ^(15)^ in 1970. Later, departments of genetics were established in several children’s hospitals in Shizuoka, Saitama, Chiba, and Nagano, but were limited to clinical genetics medical care in the field of pediatrics or perinatal care and did not lead to the spread of clinical genetics services covering other areas, such as late-onset genetic diseases, familial cancers, susceptibility of monogenic or polygenic diseases, and so on.

After the establishment of the Division of Clinical and Molecular Genetics (currently the Center for Medical Genetics), Shinshu University Hospital in 1996 ^(16)^, cross-disciplinary diagnosis and therapy departments were established one after another, primarily in university hospitals. In other words, departments of clinical practice of medical genetics that could handle genetic testing and genetic counseling not only in pediatric and perinatal fields, but also in other domains, including familial cancers, hereditary neurological disorders, hereditary deafness and other various genetic diseases, were established one after another. In addition, the following description significantly influenced the ethical guidelines for human genome and gene analysis and research ^(17)^ in 2001: “If the researcher in-charge tries to disclose genetic information about a single genetic disease, it is important to adequately consider the medical or psychological consequences and disclose the information in close collaboration with the physician in-charge of the diagnosis and treatment, and apart from this, if required, opportunities for genetic counseling must be provided. Genetic counseling should be conducted in collaboration with doctors and medical professionals who have sufficient knowledge of medical genetics and are proficient in genetic counseling.”

In Japan, because conducting genetic counseling in various fields is considered essential for conducting research in genomic medicine, it can be said that a system for cross-disciplinary clinical practice of medical genetics can be developed for each department depending on the voluntary efforts of each university and medical care institution.

## IV. The National Liaison Council for Clinical Sections of Medical Genetics (NLCCSMG, 2003-) ^(18)^

The National Liaison Council for Clinical Sections of Medical Genetics was established in 2003 with the aim of ensuring cooperation among sections of clinical practice of medical genetics, including university hospitals and other medical institutions, as well as to contribute to the development of clinical practice of medical genetics (such as genetic counseling and genetic testing) through the exchange of information related to academic and social matters and through the mutual exchange of opinions among members. The requirements for becoming a support institution member of the organization include any of the following criteria:

a. A medical educational institution, such as a university hospital,

b. a national institute for advanced medical care,

c. The Japanese Board of Medical Genetics and Genomics, Clinical Genetics system and training facility,

d. a hospital that is planning to become the training facility of Japanese Board of Medical Genetics and Genomics, Clinical Genetics system,

e. a facility that nurtures certified genetics counselors, or

f. a hospital that is systematically involved in clinical practice of medical genetics.

As of January 2019, a total of 117 medical institutions, including all university hospitals, had joined the NLCCSMG as support institution members. At the annual meeting of NLCCSMG, we conducted activities to summarize the various issues of Clinical Sections of Medical Genetics and the recommendations for solving them, and the results were published on the report and on this website. In addition, it conducts various activities, including the management of a retrieval system for the clinical practice of medical genetics implementation facilities, operating of comprehensive information sites related to clinical genetics medicine for specialists, and the business of renting DVDs of lecture series on medical genetics. In the future, there are plans to position the advanced medical training program Next Generation Super Doctor Project ^(19)^, which has been implemented as an auxiliary project of the Ministry of Education, Culture, Sports, Science and Technology, as an activity of this Liaison Council that will be developed as a nationwide human resource development project.

## V. The Japanese Association of Medical Sciences Guidelines for Genetic Testing and Diagnosis in Medicine ^(20)^

The Japanese Association of Medical Sciences ^(21)^ aims to promote research in science and technology related to medicine and is the highest institution in the field of medicine in Japan. It was founded in 1902 with the objective of contributing to the improvement in medicine and medical care standards. As of 2019, 129 major medical societies have joined, including the JSHG. With the JSHG at the center and with the cooperation of 16 other associations, the guidelines for genetic testing, diagnosis, and treatment in medical care were established in 2011; these guidelines has now become the standard norm for genomic medicine and medical care in Japan.

The guidelines emphasize the importance of providing sufficient and careful consideration to the characteristics of genetic information when conducting genetic testing, diagnosis, and treatment in an institution that provides medical care, as well as toward the need for implementing it properly and effectively. The guidelines describe the fundamental aspects and principles that physicians need to consider. Apart from genetic tests conducted at medical institutions or those for diagnosing and treating patients who are already suffering from symptoms of an illness, the guidelines mention the need for performing carrier testing, pre-symptomatic testing, susceptibility testing, pharmacogenetic testing, prenatal testing, neonatal mass screening testing, and others.

## VI. The Japanese Association of Medical Sciences Committee on Genetics, Health and Society ^(22)^

In the wake of the creation of the guidelines for genetic testing, diagnosis, and treatment in medical care, the Association reaffirmed the need to continue to work on the following genetic related issues: how to handle genetic information in clinical practice (which is the ideal way of genetic counseling), direct-to-consumer genetic testing, genetic testing and children, genetic testing and cancer, genetic testing of multifactorial disease, pharmacotherapy and genetics as well as in a system for quality assurance, inspection, and provision of tests.

The association also established the committee on Genetics, Health and Society that proactively conducts the following activities:

### 1) Significant concerns being expressed for the expansion of the genetic testing market ^(23)^

DNA used for genetic testing can be easily obtained from the hair, nails, and buccal mucosa of the patient or consumer, irrespective of the method of blood collection. Furthermore, it is possible to outsource genetic analysis to foreign companies, rather than be restricted to domestic companies. In fact, there are no regulations with respect to genetic testing services in Japan, and significant concerns were expressed in 2012 owing to the emergence of companies that conduct gene testing services with scarce scientific evidence as a business without going through specialized medical institutions.

### 2) Non-invasive prenatal genetic testing (NIPT) ^(24)^

Because NIPT can predict the genetic information of the fetus with considerable probability by simply collecting blood from the pregnant woman, the Japan Society of Obstetrics and Gynecology established guidelines for “new prenatal genetic tests using maternal blood,” which emphasize that NIPT can be conducted only in accredited and registered facilities where sufficient genetic counseling can be performed. Because NIPT is not confined to the field of obstetrics and gynecology, the Japan Medical Association, The Japanese Association of Medical Sciences, the Japan Society of Obstetrics and Gynecology, the Japan Association of Obstetricians and Gynecologists, and the JSHG have published a joint statement regarding new prenatal genetic tests using maternal blood stating that the guidelines of the Japan Society of Obstetrics and Gynecology should be complied with and that the accreditation and registration of facilities conducting NIPT should be conducted under the Accreditation and Registration Committee for facilities conducting the tests established by The Japanese Association of Medical Sciences Committee on Genetics, Health and Society in 2012.

### 3) Japanese Organization of Hereditary Breast and Ovarian Cancer (JOHBOC) ^(25)^

JOHBOC has its origins in the discussions held in The Japanese Association of Medical Sciences Committee on Genetics, Health and Society. JOHBOC, established in 2016, and aims to develop and expand the medical system for hereditary breast and ovarian cancer and for patients and family members suspected of the same, as well as to contribute to the improvement of national healthcare and preventive medicine with the cooperation of the JSHG, the Japanese Breast Cancer Society, and the Japan Society of Obstetrics and Gynecology. Its main projects include the accreditation of facilities for the treatment of hereditary breast and ovarian cancer, education, and training related to hereditary breast and ovarian cancer, the registration of patients with hereditary breast and ovarian cancer, and conducting research on hereditary breast and ovarian cancer.

## VII. Future Challenges

When considering the promotion of genomic medicine in Japan, it is necessary to consider the possibility that the general public may be misled by legal, administrative, ethical, and educational aspects of genomic medicine, which have not yet been adequately established. As indicated by Peter S. Harper ^(3)^, Japanese have a specific way of approaching genetics. Because of homogeneity, Japanese do not tend to like being differentiated from other groups of people and tend to think that it is better to be conspicuous in the group to which one belongs, often leading to the elimination of diversity and discrimination. Rare hereditary or genetic diseases are particularly avoided. Moreover, some people have the irrational thought that hereditary or genetic diseases are evil conditions that must be concealed for a long time even after they have been identified and that having a “bad” gene is someone’s fault ^(26)^.

To overcome this situation and realize the power of genomic medicine, the following three points should be considered: 1) To establish a genetic counseling system so that anyone can receive genetic counseling whenever and wherever necessary, 2) To promote a proper understanding of “genetics” to the general public, and 3) To frame enforceable laws, regulations, and policies to prevent the inappropriate use of genetic information.

### 1) Genetic counseling system

As introduced in “IV. The National Liaison Council for Clinical Sections of Medical Genetics,” genetic counseling systems are being established in various parts of the country, primarily university hospitals, by the voluntary efforts of members of associations related to medical genetics. However, genetic counseling is reimbursed by the Japanese governmental health insurance for only 79 genetic diseases, moreover, only in the case where genetic testing is performed. It will be necessary to treat genetic counseling as an independent specialized medical practice rather than as a medical examination fee, which means the cost of genetic counseling should be paid from National Health Insurance, especially in the case of genetic counseling conducted at the hospital with Clinical Sections of Medical Genetics where clinical geneticists and certified genetic counselors are always working.

### 2) Promoting a proper understanding of “genetics” to the general public

Medical genetics associations in Japan have made various efforts to promote a proper understanding of genetics. Here are some examples:

- A workshop offered to elementary, junior high, and high school teachers: How to teach human “genetics” and “diversity” in primary and secondary curriculum ^(27)^
- Science cafe for middle and high school students: Carrier genetic testing—Do you want to know about yourself, your partner, and to what extent ^(28)^?
- Joint symposium by geneticists and media personnel: What does society ask the media? What does the media ask society ^(29)^?
- Open public lecture: Genetic disease? Intractable disease? What should I do ^(30)^?
- Publication of reference materials on human genetics for high school biology education ^(31)^
- Publication of the Medical Genetics Education Model Curriculum (2013) ^(32)^
- By this initiative, the item of “clinical genetics practice and genomic medicine” was added to the medical education model core curriculum defined by MEXT for the first time in 2017 ^(33)^.

As described in the philosophy and future plan of the JSHG ^(5)^, in view of the fact that inter-individual diversity is becoming clear in detail, we have to be careful to ensure that the differences among these individuals do not lead to discrimination. The medical genetics associations in Japan will continue to actively carry out education and social enlightenment activities in order to build a society in which all are respected on the premise of human diversity.

### 3) Preventing inappropriate use of genetic information

In the United States, the Genetic Information Nondiscrimination Act and Health Insurance Portability and Accountability Act enforce the proper use of personal genetic information.

However, recently, it has been reported that companies that provide genetic testing services to investigate ancestry also provide police authorities with customers’ genome information and cooperate in criminal investigations. The debate on the protection of personal genetic information and its use in society continues ^(34)^.

There is a personal information protection Act (Kojin Joho Hogo Ho) in Japan that addresses the protection of personal genetic information. However, it is impossible to appropriately use genetic information only by this Act. Currently, multiple direct-to-consumer (DTC) DNA analysis businesses are rampant as DNA analysis is not considered medical practice in Japan ^(35)^. In addition to genetic testing in clinical practice and genome analysis for research purposes, appropriate legal arrangements covering DTC genetic testing are essential not only to prevent genetic discrimination, but also to promote genomic medicine.

## Conclusion

At present, the promotion of genomic medicine has become a national policy in Japan and specific measures are being implemented to achieve this objective. However, it remains essential to support the system created by the grassroots movements of academia described herein.

## Article Information

### Conflicts of Interest

None

### Sources of Funding

This work was partly supported by “Cancer Control Promotion Project” from the Ministry of Health, Labour, and Welfare [grant number: H29-gantaisaku-ippan-003].

### Acknowledgement

We would like to thank Dr. Yoichi Matsubara, President of the JSHG, and all members of the society for providing valuable information.

The authors would like to thank Enago (www.enago.jp) for the English language review.
